# Supplementary material for: Parasitic Infections in Stranded Whales and Dolphins in Canary Islands (2018–2022): An Update
Source: Animals (Basel). 2024 Nov 23;14(23):3377. doi: 10.3390/ani14233377 (PMC11640159; doi:10.3390/ani14233377)
Supplement: Supplementary file 1 [file animals-14-03377-s001.zip › animals-3314908-supplementary.pdf]

Table S1. Biological data and stranding locations of 233 stranded and necropsied cetaceans. Sex: female (F), male (M), not determined (ND). Age: neonate (N), calf (C), juvenile–subadult (J), adult (A). Type of stranding (TS): dead (D), alive (Al). Body condition (BC): good (G), moderate (Md), poor (P), very poor (VP), not determined (ND). Conservation status (CS): very fresh (VF), fresh (F), moderate autolysis (MA), advanced autolysis (AA). Stranding location, islands (IS): Gran Canaria (GC), Fuerteventura (FV), Lanzarote (LZ), Tenerife (TF), La Gomera (LG), El Hierro (EH), La Palma (LP), La Graciosa (LGra).

No	Specie	Sex	Age	TS	BC	CS	IS
CET 881	*Stenella coeruleoalba*	M	J	D	P	MA	FV
CET 882	*Stenella frontalis*	M	C	D	ND	AA	FV
CET 883	*Kogia breviceps*	M	J	D	Md	AA	FV
CET 884	*Stenella frontalis*	F	C	D	Md	Fr	TF
CET 885	*Stenella frontalis*	M	A	D	Md	MA	FV
CET 886	*Stenella coeruleoalba*	F	C	D	Md	AA	FV
CET 887	*Kogia breviceps*	F	J	D	P	Fr	TF
CET 888	*Steno bredanensis*	M	J	D	Md	AA	GC
CET 889	*Delphinus delphis*	F	A	D	Md	Fr	LZ
CET 891	*Stenella coeruleoalba*	M	A	D	Md	MA	GC
CET 892	*Stenella coeruleoalba*	M	J	D	Md	MA	TF
CET 894	*Steno bredanensis*	M	N	D	P	Fr	GC
CET 895	*Steno bredanensis*	F	A	D	P	MA	GC
CET 896	*Stenella coeruleoalba*	F	N	D	P	Fr	GC
CET 897	*Stenella coeruleoalba*	M	N	D	P	AA	TF
CET 898	*Stenella coeruleoalba*	F	C	Al	Md	Fr	LZ
CET 899	*Tursiops truncatus*	F	J	D	P	Fr	GC
CET 900	*Steno bredanensis*	F	A	Al	VP	VF	GC
CET 903	*Tursiops truncatus*	F	A	D	VP	Fr	TF
CET 904	*Delphinus delphis*	M	N	D	ND	MA	GC
CET 905	*Delphinus delphis*	F	A	D	Md	MA	LGra
CET 906	*Delphinus delphis*	M	N	D	Md	Fr	FV
CET 907	*Stenella coeruleoalba*	M	C	D	P	Fr	GC
CET 908	*Balaenoptera acutorostrata*	M	A	D	P	AA	LG
CET 909	*Globicephala macrorhynchus*	F	A	D	ND	AA	EH
CET 911	*Physeter macrocephalus*	M	C	D	ND	AA	LZ
CET 912	*Stenella frontalis*	M	N	D	Md	Fr	TF
CET 914	*Ziphius cavirostris*	F	A	D	VP	AA	LZ
CET 915	*Stenella coeruleoalba*	M	A	D	ND	AA	EH
CET 916	*Kogia breviceps*	M	J	D	ND	AA	LP
CET 917	*Stenella frontalis*	F	A	D	ND	AA	TF
CET 918	*Stenella frontalis*	F	C	D	ND	MA	TF
CET 920	*Globicephala macrorhynchus*	M	A	Al	P	Fr	GC
CET 921	*Stenella coeruleoalba*	M	J	D	P	MA	GC
CET 923	*Delphinus delphis*	F	J	D	ND	AA	FV
CET 925	*Kogia breviceps*	M	A	D	ND	AA	FV
CET 926	*Stenella coeruleoalba*	M	J	D	Md	MA	FV
CET 927	*Kogia sima*	F	A	D	ND	AA	LZ
CET 929	*Kogia sima*	M	J	D	ND	MA	TF
CET 930	*Delphinus delphis*	M	J	D	P	Fr	FV
CET 933	*Stenella coeruleoalba*	M	J	Al	Md	Fr	GC
CET 934	*Tursiops truncatus*	F	C	D	VP	MA	GC

CET 936	*Grampus griseus*	M	ND	D	ND	AA	GC
CET 939	*Grampus griseus*	F	C	D	P	MA	GC
CET 940	*Stenella coeruleoalba*	ND	ND	D	ND	AA	GC
CET 945	*Stenella frontalis*	ND	J	D	ND	AA	LZ
CET 947	*Delphinus delphis*	M	J	D	Md	MA	FV
CET 948	*Stenella coeruleoalba*	F	C	D	G	MA	FV
CET 949	*Globicephala macrorhynchus*	M	A	D	G	MA	TF
CET 950	*Stenella coeruleoalba*	F	A	D	Md	MA	GC
CET 951	*Stenella coeruleoalba*	F	C	D	Md	Fr	LP
CET 952	*Mesoplodon densirostris*	M	A	D	Md	Fr	FV
CET 953	*Physeter macrocephalus*	M	J	D	G	Fr	GC
CET 956	*Stenella coeruleoalba*	F	A	D	Md	MA	FV
CET 957	*Kogia breviceps*	M	C	D	P	MA	FV
CET 959	*Stenella coeruleoalba*	F	J	D	Md	MA	FV
CET 963	*Lagenodelphis hosei*	M	C	D	Md	MA	GC
CET 965	*Physeter macrocephalus*	F	J	Al	G	Fr	GC
CET 967	*Stenella coeruleoalba*	M	A	D	Md	Fr	FV
CET 968	*Delphinus delphis*	M	A	D	P	Fr	TF
CET 969	*Globicephala macrorhynchus*	F	C	Al	P	VF	TF
CET 974	*Balaenoptera edeni*	M	J	D	G	MA	FV
CET 976	*Physeter macrocephalus*	F	J	D	Md	MA	LP
CET 977	*Grampus griseus*	F	C	D	VP	Fr	GC
CET 979	*Physeter macrocephalus*	F	J	D	ND	AA	TF
CET 981	*Stenella frontalis*	F	J	D	ND	AA	FV
CET 982	*Delphinus delphis*	M	C	D	ND	AA	FV
CET 983	*Stenella coeruleoalba*	M	J	D	Md	Fr	GC
CET 984	*Grampus griseus*	M	J	Al	VP	VF	GC
CET 985	*Stenella coeruleoalba*	M	A	D	P	Fr	TF
CET 988	*Stenella coeruleoalba*	F	A	D	Md	MA	FV
CET 990	*Stenella coeruleoalba*	M	A	D	G	Fr	TF
CET 991	*Stenella coeruleoalba*	M	A	D	VP	Fr	FV
CET 992	*Delphinus delphis*	F	J	D	P	AA	FV
CET 993	*Stenella frontalis*	M	C	D	P	Fr	TF
CET 994	*Delphinus delphis*	F	A	D	ND	AA	FV
CET 995	*Globicephala macrorhynchus*	M	J	D	Md	Fr	GC
CET 996	*Globicephala macrorhynchus*	M	J	D	P	MA	FV
CET 997	*Stenella frontalis*	M	N	D	P	Fr	TF
CET 998	*Ziphius cavirostris*	F	ND	D	ND	AA	LZ
CET 999	*Stenella coeruleoalba*	F	C	Al	P	VF	GC
CET 1000	*Globicephala macrorhynchus*	F	A	D	ND	AA	LZ
CET 1002	*Grampus griseus*	M	C	D	VP	AA	FV
CET 1009	*Lagenodelphis hosei*	M	N	D	Md	MA	TF
CET 1012	*Mesoplodon europaeus*	M	ND	D	ND	AA	LP
CET 1014	*Stenella coeruleoalba*	M	A	D	Md	Fr	GC
CET 1016	*Stenella coeruleoalba*	M	N	D	P	Fr	LP
CET 1019	*Mesoplodon europaeus*	F	C	D	Md	MA	EH
CET 1020	*Tursiops truncatus*	F	J	D	Md	Fr	TF
CET 1022	*Globicephala macrorhynchus*	F	A	D	ND	AA	TF
CET 1025	*Stenella coeruleoalba*	F	J	Al	Md	Fr	LZ
CET 1026	*Stenella frontalis*	M	A	D	ND	AA	LZ
CET 1027	*Ziphius cavirostris*	M	J	D	Md	Fr	TF
CET 1031	*Stenella coeruleoalba*	M	A	D	Md	MA	TF
CET 1032	*Stenella coeruleoalba*	F	A	Al	P	Fr	TF

CET 1033	*Grampus griseus*	F	C	D	Md	MA	FV
CET 1034	*Stenella coeruleoalba*	F	J	Al	P	Fr	FV
CET 1035	*Stenella coeruleoalba*	F	J	Al	P	Fr	FV
CET 1038	*Stenella coeruleoalba*	M	A	Al	P	Fr	LZ
CET 1040	*Globicephala macrorhynchus*	F	A	D	ND	Fr	TF
CET 1042	*Tursiops truncatus*	M	A	D	P	Fr	TF
CET 1044	*Stenella frontalis*	F	C	D	Md	MA	TF
CET 1045	*Delphinus delphis*	F	A	Al	P	Fr	FV
CET 1048	*Ziphius cavirostris*	M	J	Al	P	Fr	GC
CET 1049	*Kogia breviceps*	F	C	Al	Md	Fr	FV
CET 1050	*Stenella frontalis*	M	A	D	Md	Fr	TF
CET 1054	*Stenella coeruleoalba*	F	A	D	VP	Fr	LZ
CET 1055	*Stenella coeruleoalba*	F	A	D	P	MA	FV
CET 1056	*Stenella frontalis*	M	C	D	Md	Fr	TF
CET 1057	*Stenella frontalis*	F	A	D	Md	Fr	GC
CET 1058	*Stenella frontalis*	M	C	D	P	Fr	GC
CET 1059	*Grampus griseus*	M	J	D	ND	MA	FV
CET 1062	*Balaenoptera physalus*	M	C	Al	P	Fr	FV
CET 1063	*Stenella coeruleoalba*	M	A	D	Md	Fr	TF
CET 1064	*Stenella coeruleoalba*	F	A	D	ND	AA	GC
CET 1067	*Stenella frontalis*	F	N	D	Md	Fr	TF
CET 1068	*Delphinus delphis*	F	C	D	P	MA	TF
CET 1069	*Stenella coeruleoalba*	M	A	D	P	Fr	GC
CET 1070	*Stenella frontalis*	F	N	D	Md	Fr	TF
CET 1071	*Balaenoptera acutorostrata*	F	ND	D	VP	Fr	TF
CET 1074	*Ziphius cavirostris*	M	J	D	Md	Fr	TF
CET 1089	*Stenella frontalis*	M	J	D	ND	MA	GC
CET 1090	*Globicephala macrorhynchus*	M	A	D	Md	Fr	TF
CET 1091	*Delphinus delphis*	M	C	D	P	Fr	TF
CET 1092	*Stenella coeruleoalba*	F	J	D	Md	Fr	GC
CET 1094	*Physeter macrocephalus*	F	A	D	ND	AA	GC
CET 1095	*Stenella frontalis*	F	N	D	VP	Fr	TF
CET 1099	*Delphinus delphis*	ND	N	D	ND	AA	GC
CET 1100	*Stenella frontalis*	F	A	D	Md	Fr	LP
CET 1103	*Tursiops truncatus*	M	J	Al	P	Fr	GC
CET 1104	*Stenella frontalis*	M	J	Al	Md	Fr	FV
CET 1105	*Stenella coeruleoalba*	M	A	Al	Md	Fr	FV
CET 1106	*Globicephala macrorhynchus*	F	C	D	ND	MA	LZ
CET 1107	*Stenella coeruleoalba*	ND	J	D	ND	AA	GC
CET 1110	*Stenella frontalis*	F	A	D	P	Fr	FV
CET 1114	*Stenella coeruleoalba*	M	N	D	P	Fr	TF
CET 1118	*Stenella frontalis*	F	A	Al	P	Fr	LP
CET 1121	*Tursiops truncatus*	M	A	D	ND	AA	GC
CET 1122	*Stenella frontalis*	M	A	Al	Md	Fr	FV
CET 1125	*Mesoplodon europaeus*	F	A	D	ND	AA	EH
CET 1127	*Stenella coeruleoalba*	F	A	D	Md	Fr	TF
CET 1128	*Stenella coeruleoalba*	M	A	D	P	Fr	FV
CET 1133	*Tursiops truncatus*	M	A	D	P	Fr	FV
CET 1137	*Kogia breviceps*	M	A	Al	Md	MA	TF
CET 1138	*Stenella frontalis*	M	C	D	Md	Fr	GC
CET 1139	*Kogia breviceps*	M	A	D	Md	MA	FV
CET 1142	*Mesoplodon europaeus*	M	A	D	Md	MA	FV
CET 1145	*Stenella frontalis*	M	C	D	Md	Fr	FV

CET 1146	*Delphinus delphis*	M	A	D	P	MA	TF
CET 1147	*Stenella frontalis*	F	J	D	P	MA	GC
CET 1150	*Steno bredanensis*	F	A	D	ND	AA	TF
CET 1151	*Tursiops truncatus*	M	J	D	Md	Fr	TF
CET 1152	*Stenella frontalis*	M	C	Al	Md	VF	GC
CET 1153	*Delphinus delphis*	M	A	D	P	Fr	GC
CET 1155	*Stenella coeruleoalba*	F	A	D	Md	Fr	FV
CET 1158	*Stenella frontalis*	M	A	D	G	Fr	TF
CET 1159	*Stenella coeruleoalba*	M	A	D	ND	AA	FV
CET 1160	*Delphinus delphis*	M	C	D	P	AA	GC
CET 1161	*Stenella frontalis*	M	A	D	Md	Fr	TF
CET 1162	*Stenella frontalis*	F	J	D	Md	Fr	GC
CET 1163	*Stenella frontalis*	F	A	D	Md	Fr	FV
CET 1164	*Stenella frontalis*	M	C	D	Md	AA	TF
CET 1165	*Stenella coeruleoalba*	F	N	D	VP	Fr	LZ
CET 1168	*Stenella coeruleoalba*	M	A	D	G	Fr	FV
CET 1169	*Tursiops truncatus*	F	A	D	ND	AA	LZ
CET 1170	*Mesoplodon densirostris*	F	A	D	Md	AA	FV
CET 1173	*Stenella frontalis*	F	C	D	G	Fr	TF
CET 1174	*Stenella coeruleoalba*	F	A	D	ND	AA	FV
CET 1180	*Stenella frontalis*	F	C	D	ND	MA	TF
CET 1181	*Globicephala macrorhynchus*	F	C	D	G	Fr	TF
CET 1186	*Delphinus delphis*	F	A	D	Md	Fr	FV
CET 1188	*Delphinus delphis*	F	A	D	P	MA	FV
CET 1190	*Kogia breviceps*	F	A	D	VP	MA	TF
CET 1191	*Tursiops truncatus*	M	A	D	P	AA	LG
CET 1192	*Stenella coeruleoalba*	M	A	Al	P	VF	TF
CET 1193	*Grampus griseus*	M	C	Al	VP	VF	GC
CET 1194	*Stenella coeruleoalba*	M	A	D	P	MA	FV
CET 1196	*Balaenoptera physalus*	M	J	D	ND	AA	GC
CET 1198	*Globicephala macrorhynchus*	F	C	D	G	MA	GC
CET 1199	*Stenella coeruleoalba*	F	J	D	Md	MA	FV
CET 1200	*Tursiops truncatus*	M	A	D	G	Fr	GC
CET 1201	*Delphinus delphis*	F	A	Al	G	Fr	GC
CET 1205	*Grampus griseus*	F	A	D	ND	AA	FV
CET 1206	*Stenella frontalis*	M	J	D	Md	MA	FV
CET 1208	*Stenella frontalis*	F	A	D	P	Fr	LZ
CET 1209	*Tursiops truncatus*	M	A	D	G	MA	TF
CET 1210	*Balaenoptera physalus*	M	J	D	ND	AA	GC
CET 1211	*Delphinus delphis*	M	A	D	ND	AA	FV
CET 1212	*Stenella frontalis*	F	A	D	Md	Fr	TF
CET 1213	*Grampus griseus*	F	ND	D	ND	AA	FV
CET 1214	*Delphinus delphis*	F	A	Al	P	VF	LZ
CET 1215	*Physeter macrocephalus*	ND	ND	D	ND	AA	TF
CET 1216	*Delphinus delphis*	F	J	D	P	Fr	TF
CET 1217	*Stenella frontalis*	F	A	D	G	Fr	TF
CET 1218	*Stenella frontalis*	M	C	D	VP	Fr	TF
CET 1219	*Physeter macrocephalus*	ND	C	D	P	AA	TF
CET 1220	*Stenella frontalis*	F	A	D	G	Fr	TF
CET 1221	*Delphinus delphis*	M	A	Al	Md	Fr	TF
CET 1222	*Delphinus delphis*	F	A	D	VP	MA	TF
CET 1223	*Stenella coeruleoalba*	M	A	Al	P	Fr	LGra
CET 1224	*Delphinus delphis*	M	A	Al	P	Fr	LZ
CET 1227	*Stenella coeruleoalba*	M	J	D	VP	Fr	TF

CET 1228	*Stenella frontalis*	M	A	D	ND	AA	TF
CET 1229	*Stenella frontalis*	M	ND	D	ND	AA	TF
CET 1230	*Stenella coeruleoalba*	F	C	D	P	Fr	LZ
CET 1231	*Stenella frontalis*	F	J	D	G	MA	GC
CET 1232	*Delphinus delphis*	M	A	Al	P	Fr	LZ
CET 1233	*Stenella coeruleoalba*	M	C	D	Md	AA	FV
CET 1234	*Stenella frontalis*	F	A	D	P	MA	TF
CET 1235	*Delphinus delphis*	M	N	D	P	MA	FV
CET 1236	*Kogia breviceps*	F	A	D	ND	AA	GC
CET 1239	*Tursiops truncatus*	M	J	Al	Md	VF	GC
CET 1240	*Stenella coeruleoalba*	M	A	Al	Md	MA	GC
CET 1241	*Stenella coeruleoalba*	M	A	Al	Md	Fr	GC
CET 1242	*Ziphius cavirostris*	F	A	D	ND	MA	TF
CET 1243	*Stenella frontalis*	F	A	Al	G	Fr	LG
CET 1244	*Globicephala macrorhynchus*	F	J	D	P	AA	GC
CET 1248	*Stenella frontalis*	M	N	D	P	Fr	GC
CET 1251	*Peponocephala electra*	M	J	Al	VP	Fr	TF
CET 1252	*Steno bredanensis*	F	N	D	Md	Fr	LZ
CET 1255	*Stenella coeruleoalba*	M	A	Al	P	Fr	TF
CET 1256	*Ziphius cavirostris*	F	A	D	ND	AA	EH
CET 1257	*Globicephala macrorhynchus*	M	J	D	Md	Fr	TF
CET 1258	*Grampus griseus*	F	C	D	P	Fr	FV
CET 1259	*Stenella coeruleoalba*	F	J	D	P	MA	FV
CET 1260	*Delphinus delphis*	M	A	Al	VP	VF	GC
CET 1261	*Tursiops truncatus*	F	A	D	VP	Fr	TF
CET 1263	*Tursiops truncatus*	F	ND	D	ND	AA	GC
CET 1264	*Tursiops truncatus*	F	A	D	Md	MA	GC
CET 1265	*Stenella frontalis*	F	A	Al	Md	Fr	LGra
CET 1266	*Stenella coeruleoalba*	M	A	Al	G	VF	LZ
CET 1268	*Ziphius cavirostris*	M	A	D	G	Fr	GC
CET 1269	*Stenella frontalis*	F	J	D	Md	MA	GC
